# Role of clinical bioinformatics in the development of network-based Biomarkers

**DOI:** 10.1186/2043-9113-1-28

**Published:** 2011-10-24

**Authors:** Xiangdong Wang

**Affiliations:** 1 Biomedical Research Center, Department of Respiratory Medicine, Fudan University Zhongshan Hospital, China

**Keywords:** protein interaction, biomarkers, clinical, disease bioinformatics

## Abstract

Network biomarker as a new type of biomarkers with protein-protein interactions was initiated and investigated with the integration of knowledge on protein annotations, interaction, and signaling pathway. A number of methodologies and computational programs have been developed to integrate selected proteins into the knowledge-based networks via the combination of genomics, proteomics and bioinformatics. Alterations of network biomarkers can be monitored and evaluated at different stages and time points during the development of diseases, named dynamic network biomarkers. Dynamic network biomarkers should be furthermore correlated with clinical informatics, including patient complaints, history, therapies, clinical symptoms and signs, physician's examinations, biochemical analyses, imaging profiles, pathologies and other measurements.

## 

Disease is a disordered or incorrectly functioning cells, tissue, organ, or system of the body, involved in multiple proteins, cells, organs and systems with the complexity. There still remains the poor understanding of molecular mechanisms by which diseases occur, even though biotechnologies and knowledge on diseases have been improved tremendously. Variations of protein-based biomarkers appear on basis of applications, e.g. functional neuro-imaging biomarkers can play in detecting, diagnosing, assessing treatment response and investigating neurodegenerative disorders [1], which may why the emphasis of much recent work has shifted to network-based biomarkers. The most of preclinical and clinical studies measure systemic levels of one or a few inflammatory proteins as an indicator of pathological alterations or disease severity, while molecular network-based approaches can describe associations between network properties, disease biology and capacity to distinguish between prognostic categories. It was suggested that information encoded in a network of inflammation proteins could predict clinical outcome after myocardial infarction [2].

Biomarkers can be gene-, protein-, peptide-, chemical- or physic-based variables. Of those biomarkers, gene- and protein-based ones have been focused and explored mostly from a single gene or protein to multiple genes or proteins, from the expression to functional indication, and from the network to dynamic network, in order to understand a multi-factorial basis responsible for the pathogenesis of diseases. Protein-protein interactions play a central and critical role in many biological functions, mediating the signaling pathways. Network biomarker as a new type of biomarkers with protein-protein interactions was initiated and investigated with the integration of knowledge on protein annotations, interaction, and signaling pathway. It was found that network biomarkers discovered on basis of protein knowledge on the SELDI-TOF-MS data were better than single biomarkers without any protein-protein interaction in patient classification [3].

A number of methodologies and computational programs have been developed to integrate selected proteins into the knowledge-based networks via the combination of genomics, proteomics and bioinformatics. Those methodologies include gene regulatory network inference tool (GRNInfer), gene regulatory network reconstruction tool with compound targets (nGNTInfer), inferring transcriptional regulatory networks from high-throughput data (nTRNInfer), inferring protein-protein interactions by parsimony principle (nInferPPI), inferring protein-protein interactions based on multi-domain cooperation (nMDCinfer), molecular network aligner (nMNAligner), detecting drug targets in metabolic networks by integer linear programming (nMetaILP), protein structure alignment tool based on multiple objective optimization (nSamo), annotating genes with positive samples (nAGPS), parsimonious tree-grow method for haplotype inference (nPTG), identifying differentially expressed pathways via a mixed integer linear programming model (nMILPs), protein-RNA binding-site prediction (nPRNA), or network ontology analysis (nNOA). Those have the own advantages and strength on basis of scientific needs and investigative goals. However, there is still a great need to validate those according to clinical application, translate those into the development of disease-specific biomarkers, and clarify the exact force of protein-protein interactions.

Furthermore, alterations of network biomarkers can be monitored and evaluated at different stages and time points during the development of diseases, which is named dynamic network biomarkers. This will provide a three dimensional imaging of protein-protein interactions to demonstrate the location and time of altered proteins, interactions or regulations in the network. Dynamic network biomarkers not only show higher or lower expression of genes or proteins, but also time-dependent stronger or weaker interactions between genes or proteins. It has considered as one of powerful ways to detect the bifurcation of gene or protein interactions, indicating the early change of biomarkers and predicting the occurrence of diseases. One of the most challenges is to translate biomarkers into clinical application and validate the disease specificity. Dynamic network biomarkers have the advantage of demonstrating pathophysiological changes at different stages and periods. The disease specificity of dynamic network biomarkers was validated by the integration with clinical informatics which translates clinical descriptive information on complaints, sign, symptoms, biochemical analyses, imaging and therapies into the digital data [4]. Comparing dynamic alterations of network biomarkers with clinical informatics may allow us to discover disease-specific, stage-specific, severity-specific or therapy-sensitive biomarkers.

Clinical bioinformatics has been suggested as a new emerging science combining clinical informatics, bioinformatics, medical informatics, information technology, mathematics and omics science together [5]. Clinical bioinformatics was initially proposed to enable researchers to search online biological databases, use bioinformatics in the medical practice, select appropriate software to analyze the microarray data for medical decision-making, and optimize the development of disease-specific biomarkers and supervise drug target identification and clinical validation [6]. Understanding the interaction between clinical informatics and bioinformatics is the first and critical step to discover and develop the new diagnostics and therapies for diseases. In order to optimally select and validate the disease specificity and clinical values, dynamic network biomarkers should be furthermore correlated with clinical informatics, including patient complaints, history, therapies, clinical symptoms and signs, physician's examinations, biochemical analyses, imaging profiles, pathologies and other measurements. There is a great need for scientific channels and tools to bridge clinical bioinformatics to the development, standardization, application and optimization of selected dynamic network biomarkers.

There is a real challenge to translate dynamic network biomarkers into the understanding of clinical symptoms and signs, disease development and progress, and therapeutic strategy. Networks of genes and proteins generated from computational program on basis of knowledge present the links and association between, while such knowledge-integrated interaction is relatively defined and fixed. However, it is expected that the strength of interactions between genes or proteins should be varied during the development of diseases, rather than only the expression. It is also important to clarify whether the functional correlation exists between networks of genes and proteins, network biomarkers differ from dynamic network biomarkers, there is clinical relevance and correlation between dynamic network biomarkers and clinical informatics, or we can understand molecular mechanism of diseases better from dynamic network biomarkers. In order to reach clinical application, the advantages and disadvantages of protein-based network biomarkers should be furthermore investigated to evaluate the potential values of network biomarkers in the development. Thus, we believe that clinical bioinformatics can play an important role in identification and validation of disease-specific dynamic network biomarkers.
